# Indents

**Published:** 1911-10-15

**Authors:** 


### INDENTS.

**☞Brief Articles of Technical or Scientific Value will receive notice and comment. Contributions invited.**

*Plans for the Evans Museum and Dental Institute.* The building as designed will have a facade of 246 feet on Spruce street, the front being pleasingly broken by a tower to be known as the Evans tower, in the middle. Through this will be an entrance to all parts of the building. The Evans Museum, where will be exhibited all the trophies of the late distinguished dentist, will be on the first floor at the corner of Fortieth and Spruce streets, and will be provided with a separate entrance, so that visitors may enter without disturbing any of the other occupants. The ground plan shows two long buildings, one on Spruce street and the other on Irving street, connected in the middle by another building and at the west end by another. There will be a western court and an open court on Fortieth street. This will be barred by a high ornamental railing. The depth of the lot on Fortieth street is 175 feet, and the additional room on Spruce street has been obtained by the purchase of the adjoining house, one of the pair which contained the Evans homestead. The building will be three stories in height, and the central tower will rise to a height of 100 feet. It will contain the dental museum belonging to the school and the library, also a part of the dental school. On the Irving street side will be located the dental clinic. On the third floor will be the laboratories, although there will be smaller laboratories on the first floor. Red brick with Indiana limestone trimmings will be used in the construction, which will require a year and a half to complete. The estimated cost is $800,000.—*Philadelphia Ledger.*

